# Identification of Wheat *LACCASEs* in Response to *Fusarium graminearum* as Potential Deoxynivalenol Trappers

**DOI:** 10.3389/fpls.2022.832800

**Published:** 2022-03-14

**Authors:** Zhengxi Sun, Yilei Zhou, Yi Hu, Ning Jiang, Sijia Hu, Lei Li, Tao Li

**Affiliations:** Key Laboratory of Plant Functional Genomics of the Ministry of Education, Jiangsu Key Laboratory of Crop Genomics and Molecular Breeding, Collaborative Innovation of Modern Crops and Food Crops in Jiangsu, Jiangsu Key Laboratory of Crop Genetics and Physiology, College of Agriculture, Yangzhou University, Yangzhou, China

**Keywords:** wheat, *TaLAC*, cell wall, lignin, DON, *Fusarium graminearum*

## Abstract

*Fusarium graminearum* (*F. graminearum*) can cause huge yield reductions and contamination of grain with deoxynivalenol (DON), and thus is one of the most problematic pathogen of wheat worldwide. Although great efforts have been paid and great achievements have been made to control the pathogens, there is still a wide gap for understanding the mechanism underlying *F. graminearum* resistance. Plant LACCASEs (LACs) catalyze the oxidative polymerization of monolignols by reinforcing cell-wall of various cell types to provide mechanical support, xylem sap transportation, and defense against pest and pathogens. To date, little has been known about LAC genes in bread wheat and their potential roles in wheat-*F. graminearum* interaction. Through systematic analysis of the genome-wide homologs and transcriptomes of wheat, a total of 95 *Triticum aestivum laccases* (*TaLACs)* were identified, and 14 of them were responsive to *F. graminearum* challenge. 3D structure modelings of the 14 TaLAC proteins showed that only TaLAC78 contains the entire activity center for oxidation and the others lack the type 1 copper ion ligand (T1Cu). Both amino acid sequence alignment and three-dimensional reconstruction after amino acid mutation showed that the loss of T1Cu is not only related to variation of the key amino acid coordinating T1Cu, but also closely related to the flanking amino acids. Significantly differential temporal expression patterns of *TaLACs* suggested that their subfunctionalization might occur. Promoter array analysis indicated that the induction of *TaLACs* may be closely associated with salicylic acid signaling, dehydration, and low-oxygen stress under *F. graminearum* infection. Molecular docking simulation demonstrated that TaLACs can not only catalyze lignin as a substrate, but also interact with DON, which may be docked into the binding position of the monolignols, where the LACs recognize substrates. The current study provides clues for exploring the novel functions of TaLACs in wheat resistance to *F. graminearum*, and TaLACs maybe candidates for conferring a high level of resistance against *F. graminearum* in wheat.

## Introduction

Fusarium head blight (FHB) is a worldwide serious wheat fungal disease that is mainly caused by Fusarium species complex (Bai and Shaner, 2004; Dean et al., 2012). FHB epidemics can cause tremendous yield losses and also have negative impacts on human health due to deoxynivalenol (DON) contamination, which is biosynthesized by Fusarium species complex such as *F. graminearum* and *F. culmorum* (Del Ponte et al., 2012; Chen et al., 2019). Hundreds of quantitative trait loci (QTL) related to FHB-resistance have been reported, and only two QTLs (*Fhb1* and *Fhb7*) were claimed to have been cloned; however, their functions remain controversial (Rawat et al., 2016; Li et al., 2019; Su et al., 2019; Wang et al., 2020; Zheng et al., 2020; Guo et al., 2021). Our knowledge of the molecular mechanism underlying wheat–*Fusarium* species interaction is still quite limited. Four types of wheat FHB resistance have been proposed, including resistance to initial pathogen infection (type I), resistance to pathogen spread within a spike (type II), resistance to toxin accumulation (type III), and resistance to kernel infection (type IV) (Miller et al., 1985; Mesterházy, 1995; Gong et al., 2020). Type II resistance is one of the main types of resistance. The combination of cell-wall composition and lignification plays a pivotal role in host resistance to FHB (Lahlali et al., 2016). Lignin is the second most abundant complex biological polymer on our planet after cellulose and an integral part of plant secondary cell wall (O’Leary, 2020). Lignin deposition in the fungus-infected cells limits the ingress of pathogens and also prevents the transfer of water and nutrients from plant to pathogen to restrict pathogen growth (Hu et al., 2018). Laccases (LACs, benzenediol oxygen reductases, EC 1.10.3.2) are necessary for lignin polymerization during secondary cell wall formation (Berthet et al., 2011; Zhao et al., 2013). However, the report of LACs in FHB resistance is scarce, and only a preliminary study has been reported (Soni et al., 2020).

LACCASEs are multicopper containing oxidases (MCOs) that widely exist in higher plants (Mayer and Staples, 2002), fungi (Brijwani et al., 2010), bacteria (Santhanam et al., 2011), lichens (Laufer et al., 2006), insects (Yamazaki, 1969; Dittmer and Kanost, 2010), and in sponges (Li et al., 2015). Typically, each LAC molecule contains four copper ions in the active core, which consists of three actively binding sites according to their spectroscopic features: type 1 (T1) with one copper ion, type 2 (T2) also with one copper ion, and type 3 (T3) with two copper atoms, an antiferromagnetically coupled binuclear copper pair (T3A and T3B) (Solomon et al., 1996). The T2 copper and T3 coppers are arranged in a trinuclear cluster (TNC). The four copper ions are constructed by ten histidines (His), one cysteine (Cys), and one leucine (Leu) or methionine (Met) (Sitarz et al., 2016). T1 copper is the active site where the oxidation of substrates takes place. Electrons are then shuttled along a pathway with cysteine and histidine residues to the TNC where is the site of oxygen reduction (Giardina et al., 2010; Sitarz et al., 2016). The first plant *LAC* gene was reported from the Japanese lacquer tree *Toxicodendron vernicifluum* (*Rhus vernicifera*) (Nitta et al., 2002). Subsequently, plant *LACs* have been proposed to be associated with biosynthesis and polymerization of lignin (Berthet et al., 2011; Zhao et al., 2013; Tobimatsu and Schuetz, 2019; O’Leary, 2020), flavonoid biosynthesis (Pourcel et al., 2005), root elongation (Cai et al., 2006), and multiple biotic and abiotic stress responses (Cai et al., 2006; Cho et al., 2014; Liu et al., 2017; Hu et al., 2018; Soni et al., 2020). In general, the plant LAC proteins consist of 500–600 amino acids (Morozova et al., 2007) and a high extent of glycosylation (Sterjiades et al., 1992). The theoretical isoelectric point (p*I*) of plant LAC proteins was between 7.0 and 9.6 (Morozova et al., 2007; Frasconi et al., 2010; Santhanam et al., 2011).

The conserved function of plant LACs in the cell-wall formation (Janusz et al., 2020) is widely accepted, and cell wall is the key physical barrier for a plant to resist pathogen invasion. Therefore, we deduced that LACs may play an important role in resistance to *F. graminearum*. To understand if the LACs were involved in the resistance of wheat to FHB, we systematically analyzed the members of the wheat LAC family and their responses to *F. graminearum* challenge. Wheat is an allohexaploid species with complex genetic basis, which originated from three different diploid progenitors through two hybridization events (Feldman and Levy, 2005). Therefore, polyploidization, tandem duplication, and segmental duplication result in the expansion of gene family members. In the present work, 95 *TaLACs* genes were identified using the homologous gene blast based on the protein conservation, and 14 *TaLACs* were responsive to *Fusarium graminearum* invasion. Then, physicochemical properties, conserved motif, and 3D structure and expression patterns of the 14 *TaLACs* were analyzed. We then predicted the possible mechanisms of the 14 *TaLACs* underlying DON detoxification. This study provided fundamental basis for further functional study of *TaLACs* in FHB resistance.

## Materials and Methods

### Genome-Wide Identification of LACCASE Genes in *Triticum aestivum*

Firstly, the Arabidopsis and rice LAC protein sequences were retrieved and downloaded from the Arabidopsis Information Resource (TAIR) database^1^ and China Rice Data Center database^2^. Then, we blasted AtLACs and OsLACs against the genome database of wheat Ensemble Plants database^3^ and downloaded all of the homologous genes in *Triticum aestivum*. The UniProt Knowledgebase^4^ was used to search all of the LAC protein sequences of Arabidopsis, rice, and wheat. The protein sequences identified by both methods mentioned above were integrated and parsed by manual editing to remove the redundant. The conserved domain of all the identified LACs was analyzed by blasting against the Pfam database^5^. The chromosomal distributions of the *TaLACs* were mapped by MapChart tool (2.23 version).

### Plant Materials and *F. graminearum* Inoculation

An FHB-susceptible “Chinese Spring (CS)” and an FHB-resistant variety “Sumai 3 (SM)” were used in this study. SM is a famous variety, which carries *Fhb1* conferring Type 2 resistance to FHB. Three seeds were sown in a plastic pot (10 cm × 10 cm × 8.5 cm) containing a mix of vermiculite and soil in the ratio 1:3. Thinning was carried out to accommodate one seedling per pot after 5 days of germination. The seedlings were grown in phytotron for 3 weeks under 16 h of light at 25°C and 8 h of dark at 15°C, and then were moved to a refrigerator at 4°C for vernalization for 15 days, and the seedlings were then moved back to the phytotron for growth. At anthesis, wheat spikes were inoculated with macroconidial spores of *F. graminearum* strain PH-1, which was kindly donated by Dr. Bing Li at Zhengzhou University, China. Macroconidia was produced in mung bean broth following the published protocol (Bai and Shaner, 1996). Briefly, 40 g of mung beans was placed in a 1-l Erlenmeyer flask containing 1 l of boiling deionized H_2_O. After 10 min of boiling, the mung bean broth was filtered through a cheesecloth. A total of 50 mL of the mung bean filtrate was equally subdivided into 200 mL-Erlenmeyer flask and autoclaved. The cooled mung bean filtrate was inoculated with a 2-mm^2^ disk from a culture of *F. graminearum* grown on PDA and placed on a shaker at 25°C for 4 days as spore suspension. For each wheat genotype, 10 μL of the spore suspension (100 conidia μL^–1^) was injected into the two bilateral florets of the fifth spikelet from the bottom of a spike. To collect samples at different time points, eight independent ears of wheat were inoculated with *F. graminearum*, and an additional eight ears were inoculated with mung bean broth as mock. Plants were grown in a condition-controlled phytotron under 28°C and 16 h of light/8 h of dark cycle. The inoculated spikelets and their adjoined rachis were sampled at 12 h, 1–7 days post *F. graminearum* (CSI and SMI) and mock inoculations (CSM and SMM), respectively. Six independent biological replicates were conducted with three biological replicates for RNA-sequencing and the remaining three for validation.

### mRNA Extraction and Sequencing

Spike tissues collected from the eight time points were pooled for RNA extraction. Total RNA of each treatment was extracted using RNAiso plus reagent (TAKARA BIO INC., Shiga, Japan) according to the manufacturer’s instructions. The quantification of the total RNA was performed using NanoDrop 2000c spectrophotometer (Thermo Fisher Scientific, Lenexa, KS, United States), and RNA quality was assessed using Agilent 2100 bioanalyzer (Thermo Fisher Scientific, Waltham, MA, United States). Libraries were generated and sequenced on BGISEQ-500 platform (BGI-Shenzhen, China). The sequence data was filtered with SOAPnuke (v1.5.2) (Li et al., 2008). The clean reads were mapped to the *Triticum aestivum* reference genome (Ensembl Plants 47 release, see text footnote 3) using HISAT2 (v2.0.4) (Kim et al., 2015). Bowtie2 (v2.2.5) was applied to align the clean reads to the reference gene and then the expression level of a gene was calculated by RSEM (v1.2.12) (Li and Dewey, 2011).

### Promoter Analysis

The upstream 1,500 base pair (bp) genomic DNA sequences from the transcription start sites of these *TaLAC* genes were extracted from wheat reference genome IWGSC_v1.1, and then were submitted to a database of PlantCARE^6^ to identify the putative *cis-*regulatory elements. Heatmap was drawn using TBtools software (Chen et al., 2020).

### Public Expression Profiles Extraction

Public transcriptome data were used to analyze the expression of 17 *TaLACs* under different biotic stresses from the Hexaploid Wheat Expression Database (IWGSC Annotation v1.1) in the WheatOmics (1.0) website^7^. The items of FHB stress, powdery mildew pathogen stress, stripe rust pathogen stress, and elicitation with PAMPs were chosen for further comparisons. The data was collected from the published work (Zhang et al., 2014; Biselli et al., 2018; Steuernagel et al., 2018).

### Powdery Mildew Inoculation

*Blumeria graminis f. sp. tritici* (*Bgt*) conidia was collected from the infected wheat leaves in the field. *Bgt* conidia was inoculated onto the heading-stage flag leaves, and uninoculated flag leaves were used as mock. The number of colonies was counted on the inoculated leaves of CS and SM at 10 days post-inoculation. The symptomatic and mock leaves were frozen immediately in liquid nitrogen after harvest and stored at −80°C for RNA extraction. The test was carried out with three biological replicates.

### Analysis of Physical and Chemical Property of Protein

The computations of the theoretical p*I* (isoelectric point) and Mw (molecular weight) were performed using the Compute p*I*/Mw tool on the ExPASy server^8^ (Bjellqvist et al., 1993, 1994; Wilkins et al., 1999). The potential glycosylation sites of the proteins were predicted using NetNGlyc and NetOGlyc server v1.1^9^ (Emanuelsson et al., 2000). CELLO v.2.5: subCELlular LOcalization predictor^10^ was used to predict the subcellular location of proteins (Yu et al., 2004, 2006).

### Phylogenetic Tree Construction and Conserved Motifs Analysis

A phylogenetic tree was constructed by the neighbor-joining (NJ) method with 1,000 bootstrap replicates using MEGA 7.0 and integrated with gene expression profiles *via* EvolView (V3) platform (Kumar et al., 2016; Subramanian et al., 2019). The conserved motifs for TaLAC proteins were analyzed using the Multiple Em for Motif Elicitation (MEME) server v5.3.3^11^ (Bailey et al., 2009) with a minimum and a maximum weight of 20 and 50 amino acid residues, respectively.

### Semiquantitative and Real-Time Fluorescent Quantitative PCR

A semiquantitative reverse-transcriptase polymerase chain reaction followed the published protocol (Chen et al., 1999). qRT-PCR was performed on a ABI7500 System with SYBR Premix Ex Taq (Takara, Dalian, China). The relative expression changes were calculated using the 2^–ΔΔCT^ method (Livak and Schmittgen, 2001). The constitutively expressed “housekeeping” gene *TaACTIN* was used as a reference gene. All primer sequences are listed in Supplementary Table 4.

### Prediction of Protein 3D Structure Model

The 3D structure models were built *via* a SWISS-MODEL platform^12^ based on the target-template alignment using ProMod3 (Studer et al., 2021). A template search with BLAST and HHblits was performed against the SWISS-MODEL template library. Crystal structure of the *Zea mays* laccase 3 was the reference template (Xie et al., 2020). The global and per-residue model quality were assessed using the QMEAN scoring function by homology to the model when the following criteria are met: (a) the ligands are annotated as biologically relevant in the template library, (b) the ligand is in contact with the model, (c) the ligand is not clashing with the protein, (d) the residues in contact with the ligand are conserved between the target and the template (Studer et al., 2021). If any one of these four criteria is not satisfied, a certain ligand will not be included in the model. The quaternary structure annotation of the template is used to model the target sequence in its oligomeric form. The method is based on a supervised machine learning algorithm, Support Vector Machines (SVM), which combines interface conservation (Bertoni et al., 2017), structural clustering, and other template features to provide a quaternary structure quality estimate (QSQE). The QSQE score is a number between 0 and 1, reflecting the expected accuracy of the interchain contacts for a model built based a given alignment and template. Higher numbers indicate higher reliability. This complements the GMQE score, which estimates the accuracy of the tertiary structure of the resulting model. The resulting PDB files were viewed using PyMOL (Delano, 2002).

### Molecular Docking of TaLACs With Lignin Model Compound and Deoxynivalenol

The molecular docking tool AutoDock (Morris et al., 2009) was used for the docking of sinapyl alcohol and DON on TaLAC protein. The structures of sinapyl alcohol and DON were retrieved from the ZINC database^13^. First, polar hydrogen was added on the ligand (sinapyl alcohol and DON) and TaLAC protein. To determine the probable binding site, blind docking was performed. The TaLAC protein was kept rigid, while the ligand was kept flexible to allow for the exploration of probable binding sites. AutoDock produced 10 docking conformations and ranged them according to binding energy. The conformation with the lowest binding energy was chosen for analysis, and intermolecular interactions were determined using PyMOL (Delano, 2002).

### Statistical Analysis

The charts in this study were drawn using GraphPad Prism 5 (GraphPad Software, La Jolla, CA, United States) and SigmaPlot 10.0 (Systat Software, Chicago, IL, United States). SPSS (Version 19.0, IBM) was used for data analysis. The means and standard errors of all results were calculated, and Student’s *t*-test was performed to generate *P*-values.

## Results

### Wheat *TaLAC* Genes and the Genes Responsive to *Fusarium graminearum* Infection

Protein homologous sequence alignment of 17 *AtLACs* from *A. thaliana* and 35 *OsLACs* from *O. sativa* against the current *Triticum aestivum* whole genome (Chinese Spring) assembly generated 95 putative *TaLACs* (Supplementary Table 1). We named these *LAC* genes *TaLAC1*–*TaLAC95* based on their locations on the respective chromosomes (Supplementary Figure 1 and Supplementary Table 2). Of the 95 *TaLACs* genes, 25 were densely localized on the short arm of chromosomes 3A, 3B, and 3D. Several genes, such as *TaLAC32*, *TaLAC33*, *TaLAC34*, *TaLAC39*, and *TaLAC40*, had tandem duplications (Supplementary Figure 1). Other gene clusters were on the short arm of chromosome 6D and long arms of chromosomes 7A, 7B, and 7D.

**FIGURE 1:**
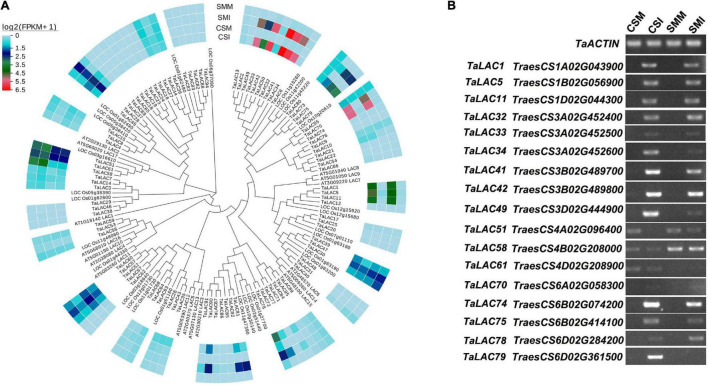
Phylogenetic tree and transcription profiles of the TaLACs family members. **(A)** The evolutionary relationship was analyzed using the Neighbor-Joining method with 1,000 bootstraps. Heat map of TaLACs expressions in CS and SM with *Fusarium graminearum* and mock inoculations were integrated in the phylogenetic tree. Log2(FPKM + 1) expression abundance was used to create the heat map. CSM, Chinese Spring inoculated with mung bean broth; CSI, Chinese Spring inoculated with *F. graminearum*; SMM, Sumai 3 inoculated with mung bean broth; SMI, Sumai 3 inoculated with *F. graminearum.* **(B)** Validation of the expressions of 17 TaLACs responsive to FHB with semi-quantitative PCR with *TaACTIN* as a reference gene.

Transcriptome sequencing shows that the majority of the *TaLACs* had low or undetectable expressions, and 17 *TaLACs* were significantly responsive to *Fusarium graminearum* infection either in CS or SM or both (Figure 1 and Supplementary Table 2). These 17 *TaLACs* were unevenly distributed on wheat genome, which were located on homologous chromosomes 1, 3, 4, and 6. Of them, three *TaLACs* on chromosome 4 were detected both in *F. graminearum*-infected spikelets and in the controls, and the other 14 *TaLACs* were detected only in *F. graminearum-*infected spikelets, which had higher expressions in CS than that in SM (Figure 1A). Interestingly, *TaLAC58* (*TraesCS4B02G20800*) had significantly higher expression in SM than that in CS (Figures 1A,B). Semiquantitative PCR confirmed the expressions of 17 *TaLACs* detected in RNA-Seq data. Thirteen of the 17 *TaLACs* were dramatically induced by *F. graminearum* challenge, but were not expressed in the controls. *TaLAC51, TaLAC58*, and *TaLAC61* were slightly inhibited by *F. graminearum* challenge, and *TaLAC70* showed weak expressions (Figure 1B).

### *Cis*-Elements in the Promoters of *TaLACs*

**FIGURE 2:**
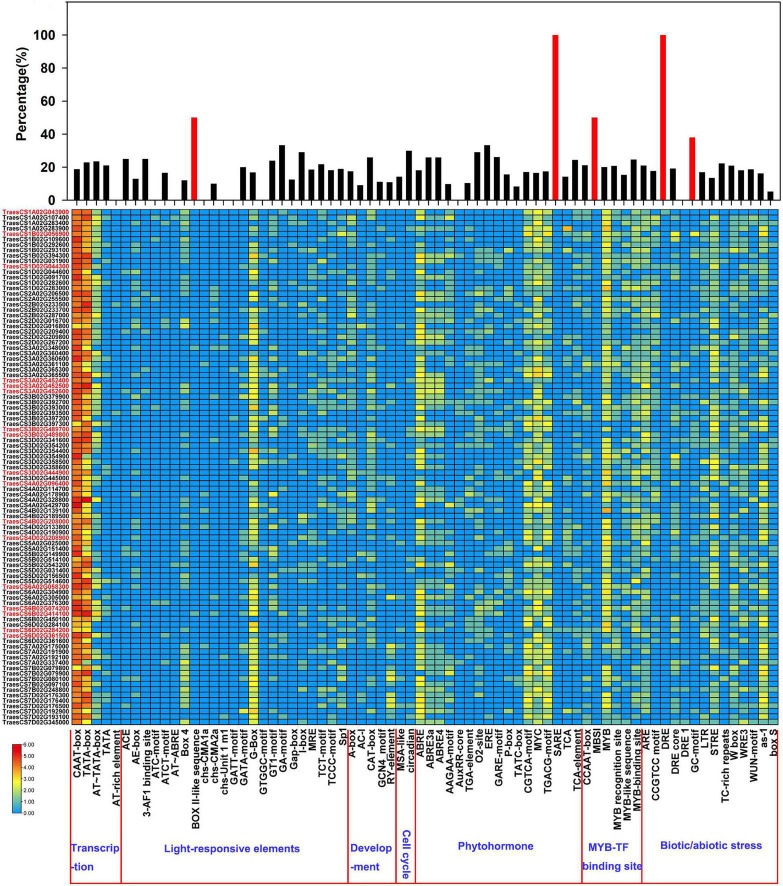
*Cis*-acting element analysis of the promoters of *TaLAC* genes. The graph was generated using *cis*-acting element numbers and functions in the promoters of TaLAC genes. Log2(NEC + 1) expressional abundance was used to create the heat map. NEC, Number of Element Copies. The upper-panel represents the ratio of the number of each *cis*-acting element in the 17 FHB responsive TaLAC genes occupying the total number of *cis*-acting elements in all of the TaLAC promoters. Red marked gene ID represent the 17 FHB responsive TaLAC genes.

The spatial and temporal expressions of genes are precisely regulated by the promoter. The 1.5-kb upstream promoter regions of the 17 *TaLACs* were predicted to explore the *cis-*regulatory elements that may be involved in response to *F. graminearum* infection. The results showed that the base-acting elements CAAT-box and TATA-box exist in the promoters of all *TaLAC* family members (Figure 2 and Supplementary Table 3). Several copy-dominant *cis-*elements were predicted in the promoters of *TaLAC* family, including light-responsive element G-box, abscisic acid (ABA) responsive element ABRE, methyl jasmonate (MeJA) responsive element CGTCA-motif, TGACG-motif, MYC transcription factor binding site, MYB transcription factor binding site, biotic or abiotic stress-responsive elements STRE, and as −1. The presence of these elements indicates that the *TaLAC* genes may be heavily involved in the light signaling pathway, the process of signal transduction of plant hormones, and the defense response to various stressors. Salicylic acid responsive element SARE and dehydration responsive element DRE specifically existed in the promoters of FHB responsive genes *TaLAC78*, *TaLAC1*, and *TaLAC11*. In addition, the total number of BOX II-like sequence, MBSI, and GC motif on the 17 FHB-responsive *TaLAC* promoters accounted for approximately one-third of the number of these elements on the 95 *TaLACs* promoters. These results suggest that the responses of the 17 *TaLACs* might be closely associated with salicylic acid signaling, dehydration, and anaerobic stress due to *F. graminearum* infection, which was consistent with the reports that salicylic acid can improve the resistance of wheat and other plants to *F. graminearum* (Makandar et al., 2010, 2012; Li et al., 2021).

### Fourteen of the 17 *TaLACs* Responded to *F. graminearum* Infection

**FIGURE 3:**
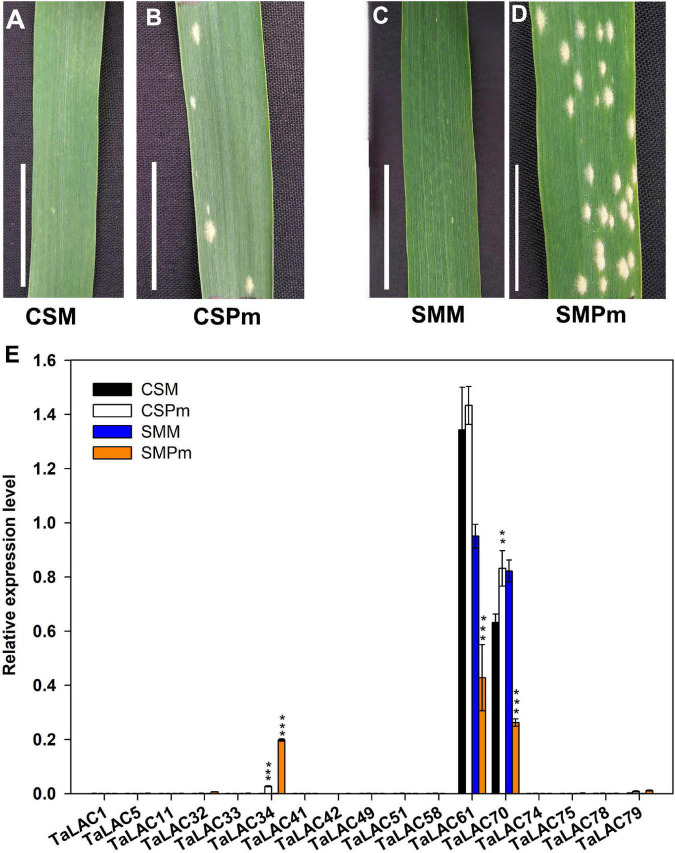
The TaLACs responsive to powdery mildew. **(A,B)** Represent the phenotype of Chinese Spring with water and powdery mildew inoculation (CSM and CSPm); **(C,D)** represent the phenotype of Sumai 3 with water and powdery mildew inoculation (SMM and SMPm). **(E)** Relative expression levels of the 17 FHB responsive TaLACs genes between wheat varieties Chinese Spring and of Sumai 3 with water and powdery mildew infections. The expression level was calculated by the 2^–ΔΔCT^ method with the wheat Actin gene as the endogenous reference for normalization. Student’s *t*-test was used to compare the difference between powdery mildew (CSPm or SMPm) and mock (CSM or SMM) inoculations of the same variety. ***P < 0.001; **P < 0.01.

To determine whether the 17 *TaLACs* were responsive to *F. graminearum* infection, the transcriptional profiles from public transcriptome data were analyzed under challenges by *F. graminearum*, powdery mildew, stripe rust, and PAMPs (Flag22 and chitin) elicitation. The expression patterns of the 17 *TaLACs* from hexaploid wheat expression database in WheatOmics 1.0 indicated that *TaLAC34* was also significantly induced by powdery mildew pathogen, but the other 16 *TaLACs* did not respond to powdery mildew infection (Supplementary Figure 2A). All the 17 *TaLACs* did not show significant change under stripe-rust challenge (Supplementary Figure 2B). Only *TaLAC34* was significantly induced in CS when challenged by Flag22 and chitin (Supplementary Figure 2C). We also analyzed the expressional profiles of the 17 *TaLACs* after being infected by *F. graminearum* according to the published transcriptome data (Biselli et al., 2018). The results showed that 14 *TaLACs* were strongly induced by *F. graminearum* in rachis or spikelet. However, *TaLAC51*, *TaLAC58*, and *TaLAC61* were inhibited by *F. graminearum* (Supplementary Figure 2D), which was highly consistent with our results. We also investigated the expressions of these 17 *TaLACs via* qRT-PCR in CS and SM leaves under powdery mildew challenge (Figure 3), which validated the transcriptome data from WheatOmics 1.0 that *TaLAC34* was dramatically induced by powdery mildew pathogen, and the expression level was higher in SM than that in CS, consistent with our observation that SM was more susceptible to powdery mildew than CS (Figure 3E). *TaLAC61* and *TaLAC70* were highly expressed in both CS and SM, but these two *TaLACs* were downregulated in SM. The other 14 *TaLACs* exhibited very low levels of expression in both CS and SM across powdery mildew pathogen infection and the controls (Figure 3E). The expression patterns were in good agreement with those available in the public database. The above results showed that *TaLAC34*, *TaLAC61*, and *TaLAC70* responded not only to *F. graminearum* but also to powdery mildew, and the remaining 14 *TaLACs* responded to *F. graminearum* infection only.

### Physicochemical Properties and Conserved Domains of the 14 Fusarium Head Blight-Responsive *TaLACs*

**FIGURE 4:**
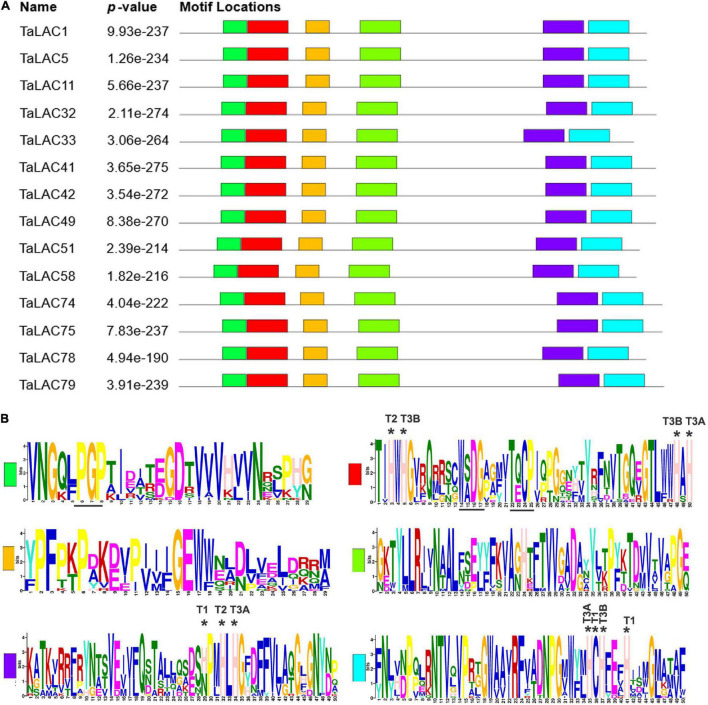
Conserved motifs from 14 TaLAC proteins specifically responsive to FHB. **(A)** Characteristic conserved domains present in the laccase proteins. The various colored boxes represent different motifs. **(B)** Sequence logo of the conserved domains. T1, T2, and T3 indicate the amino acid residue for coordinating T1, T2, and T3 copper ion, respectively. T3A and T3B represent T3 copper pair. “*” indicates the amino acid residue for coordinating copper ion.

The 14 TaLAC proteins consist of a polypeptide chain of approximately 561 (TaLAC33) to 599 (TaLAC79) amino acids and a molecular weight (MW) range from 61.7 kDa (TaLAC58) to 65.6 kDa (TaLAC75 and TaLAC79). The deduced theoretical p*I* of the 14 LACs proteins ranges from 5.8 (TaLAC5) to 8.51 (TaLAC58) through protein sequence feature analysis. All the 14 TaLACs were secreted proteins predicted to be located in periplasmic space between the cell wall and the plasma membrane (Table 1). N- or O-glycosylation sites were also predicted. Notably, six TaLAC members, including TaLAC1, TaLAC5, TaLAC11, TaLAC51, and TaLAC58 (Table 1), had more N- and O-glycosylation sites. The unique signature sequences of model plant LACs are ten histidines (His), one cysteine (Cys) and one leucine (Leu) or methionine (Met) for binding four Cu atoms, including one type-1 (T1) Cu, one type-2 (T2) Cu, and two type-3 (T3) Cu. There were six conserved motifs (Figure 4A) in the 14 FHB responsive TaLACs protein sequence, including 11 pivotal amino acid for binding Cu atoms (Figure 4B). However, we found asparagine (Asn), instead of His, present at the fifth conserved motif containing type-1 (T1) Cu across TaLAC32, TaLAC33, TaLAC41, TaLAC42, TaLAC49, TaLAC75, and TaLAC79 (Supplementary Figure 3).

**TABLE 1:** Physicochemical properties of the 14 FHB-responsive TaLACs.

Name	Gene ID	Number of amino acids	p*I*	MW (Da)	Predicted subcellular location	N-Glyc	O-Glyc
TaLAC1	TraesCS1A02G043900	577	5.96	63277.87	Periplasmic	14	9
TaLAC5	TraesCS1B02G056900	577	5.8	62981.5	Periplasmic	13	9
TaLAC11	TraesCS1D02G044300	577	5.96	62982.52	Periplasmic	14	8
TaLAC32	TraesCS3A02G452400	589	6.48	65257.32	Periplasmic	7	7
TaLAC33	TraesCS3A02G452500	561	6.2	62383.02	Periplasmic	8	2
TaLAC41	TraesCS3B02G489700	589	6.56	65392.45	Periplasmic	7	6
TaLAC42	TraesCS3B02G489800	589	6.5	65404.49	Periplasmic	6	7
TaLAC49	TraesCS3D02G444900	589	6.35	65450.42	Periplasmic	7	6
TaLAC51	TraesCS4A02G096400	569	8.33	61969.88	Periplasmic	14	6
TaLAC58	TraesCS4B02G208000	565	8.51	61731.49	Periplasmic	14	7
TaLAC74	TraesCS6B02G074200	597	5.75	65199.22	Periplasmic	8	5
TaLAC75	TraesCS6B02G414100	597	5.97	65619.06	Periplasmic	8	1
TaLAC78	TraesCS6D02G284200	577	6.6	63565.97	Periplasmic	8	5
TaLAC79	TraesCS6D02G361500	599	6.28	65600.06	Periplasmic	8	4

### Loss of T1 Copper ion in Key Amino Acid Positions in 13 Fusarium Head Blight-Responsive TaLACs

**FIGURE 5:**
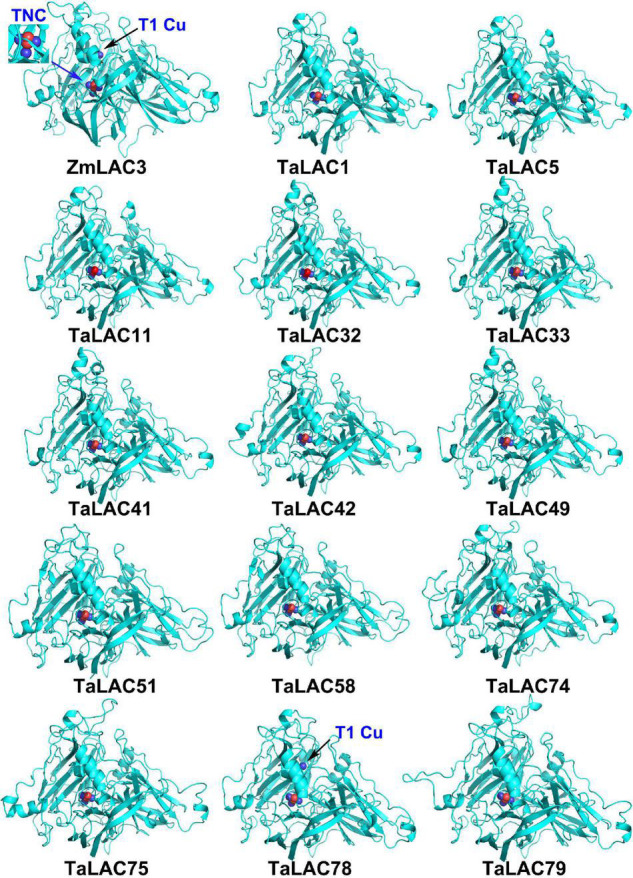
The 3D structure modeling of 14 TaLAC proteins specifically responsive to FHB. The structure image was generated *via* SWISS-MODEL platform (https://swissmodel.expasy.org/) based on the target-template alignment using ProMod3. The structures were optimized by PyMOL. Blue arrow pointed T2/T3 trinuclear copper cluster (TNC), black arrow pointed T1 copper (T1Cu). ZmLAC3 was used as the reference model. Blue sphere in the 3D protein model represents copper ion. Red sphere represents oxygen atom.

**FIGURE 6:**
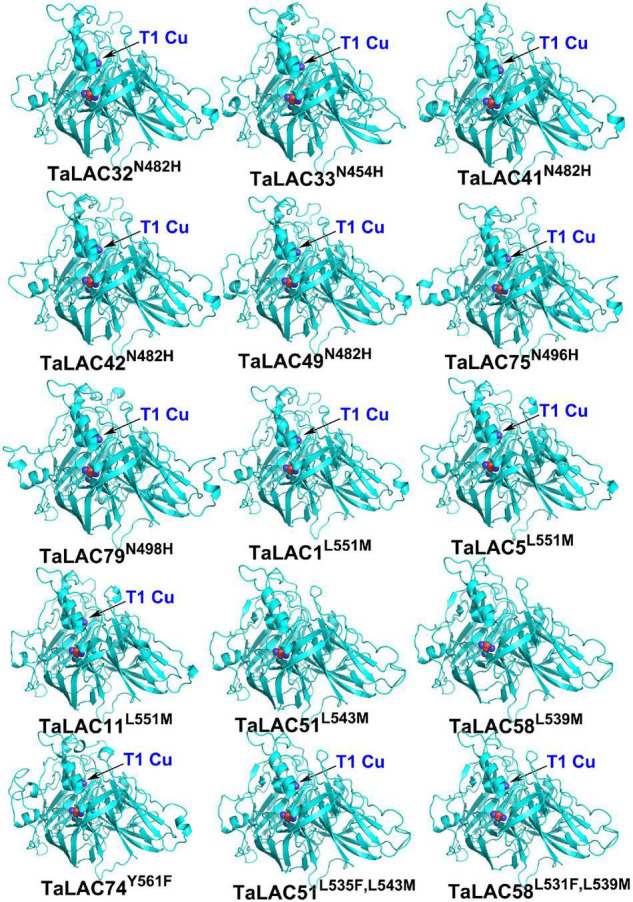
The 3D structures of mutated TaLAC proteins. The superscripts “N482H” represent replacement of asparagine (N) with histidine (H) at position 482 (N482H). The meaning of same writing style in other superscripts were the same as “N482H”. The superscripts “L535F and L543M” represent two replacements of leucine (L) with phenylalanine (F) at position 535 (L535F) and leucine (L) with methionine (M) at position 535 (L543M). The meaning of same writing style in other superscript was the same as “L535F and L543M.” The black arrow pointed T1 copper (T1Cu).

The function of a protein depends on its 3D conformation. Since the key conserved motif sequence was changed, we were wondering if the 3D structure was changed or not. 3D models of these TaLAC proteins were structured *via* ExPASy web server (SWISS-MODEL). The Global Model Quality Estimate (GMQE) scores of modeled 3D structures for the 14 TaLACs were more than 0.7 with respect to the homologous crystal structure of Laccase 3 from *Zea mays*, suggesting the prediction was reliable. The 3D structures of the 14 TaLACs were highly similar (Figure 5). All of them contain T2 copper ion, T3 copper ions, and one oxygen molecule ligand. Unexpectedly, only TaLAC78 contains T1 copper ion ligand, and the other 13 TaLACs do not have the T1 copper ion ligand (Figure 5). T1 copper ion was coordinated by two His and one Cys, but His was altered to Asn in TaLAC32, TaLAC33, TaLAC41, TaLAC42, TaLAC49, TaLAC75, and TaLAC79 (Supplementary Figure 3), which might be the main causal variation for losing the T1 copper. When Asn was changed to His *in silico* (TaLAC32^N482H^, TaLAC33^N454H^, TaLAC41^N482H^, TaLAC42^N482H^, TaLAC49^N482H^, TaLAC75^N496H^, and TaLAC79^N498H^), T1Cu ion appeared (Figure 6). To explain why TaLAC1, TaLAC5, TaLAC11, TaLAC51, TaLAC58, and TaLAC74 do not have T1 copper ion ligand despite the same residues coordinating T1 copper ion with ZmLAC3, we analyzed the 3D structure of ZmLAC3 protein and found that the T1 copper ion actually is coordinated by five residues within 4Å, including His451, Cys514, Phe(F)516, His519, and Met(M)524 (Supplementary Figure 4). We found that TaLAC1, TaLAC5, TaLAC11, TaLAC51, TaLAC58, and TaLAC74 were different from ZmLAC3 at these two sites (Supplementary Figure 3). Firstly, we simultaneously mutated Leu551 in TaLAC1, TaLAC5, and TaLAC11; Leu543 in TaLAC51; and Leu539 in TaLAC58 to Met, and the 3D models demonstrated that T1Cu ions appeared in TaLAC1^L551M^, TaLAC5^L551M^ and TaLAC11^L551M^, but neither in TaLAC51^L543M^ nor TaLAC58^L539M^ (Figure 6). Secondly, when we mutated tyrosine (Y) 561 in TaLAC74 to Phe (F), T1Cu ion appeared in TaLAC74^Y561F^, and when the two sites were mutated at the same time, T1 copper ions appeared only in TaLAC51^L535F, L543M^ and TaLAC58^L531F, L539M^ (Figure 6). These results indicate that the flanking sequences of key amino acid residue also affect the coordination of T1 copper ion ligand.

### Diverse Expression Patterns of the *TaLACs* After *F. graminearum* Inoculation

**FIGURE 7:**
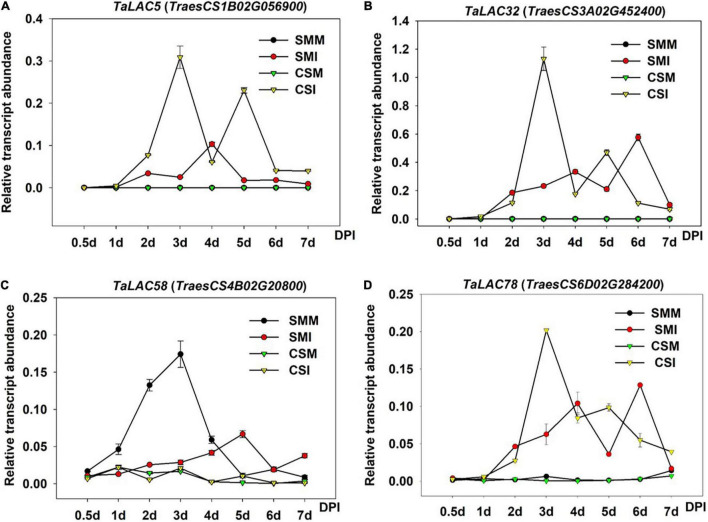
Gene expression patterns of the four TaLACs in CS and SM with Fusarium graminearum and mock inoculations across time course. **(A)** The expression pattern of TaLAC5. **(B)** The expression pattern of TaLAC32. **(C)** The expression pattern of TaLAC58. **(D)** The expression pattern of TaLAC78. DPI, days post *F. graminearum* inoculation. The expression level was calculated by the 2^–ΔΔCT^ method with the wheat Actin gene as the endogenous reference for normalization.

Since gene expression patterns provide clues for gene functions, we chose one *TaLAC* with the highest levels of expression on homologous chrs. 1, 3, 4, and 6 each to analyze the temporal expression patterns at different time points after *F. graminearum* inoculation. Differential expression patterns were observed for the *TaLACs* (Figure 7). No transcript of *TaLAC5* was detected in CS mock and SM mock, but it was induced 1 day postinoculation (DPI) with *F. graminearum*, and it reached the highest level of expression by 3 DPI in CS (Figure 7A). Interestingly, *TaLAC5* showed a second induction from 4 DPI with the peak expression at 5 DPI in CS, while it was slightly induced in SM with the highest expression level at 4 DPI (Figure 7A). *TaLAC32* and *TaLAC78* had similar expression patterns with *TaLAC5* in mock samples of the two varieties, while they were induced by *F. graminearum* infection at one DPI, and the peak expression was at 3 DPI in CS (Figure 7A), but they had a delayed peak intensity until 6 DPI in SM (Figures 7B,D).

Obviously, *TaLAC5*, *TaLAC32*, and *TaLAC78* showed similar expression patterns, and their expressions in CS were significantly higher than their counterparts in SM (Figures 7A,B,D). The expression of *TaLAC58* showed a different pattern from the other three *TaLACs*. The expression was quite weak in CS with both *F. graminearum* infection and mock inoculation, and it was inhibited until 4 DPI and a slight induction was observed at 5 DPI in SM (Figure 7C). These results implied that *TaLACs* may play different functions during the process of *F. graminearum* infection, despite a loss of oxidizing ability due to the absence of the T1 copper.

### TaLAC Could Be a Potential Deoxynivalenol Trapper

**FIGURE 8:**
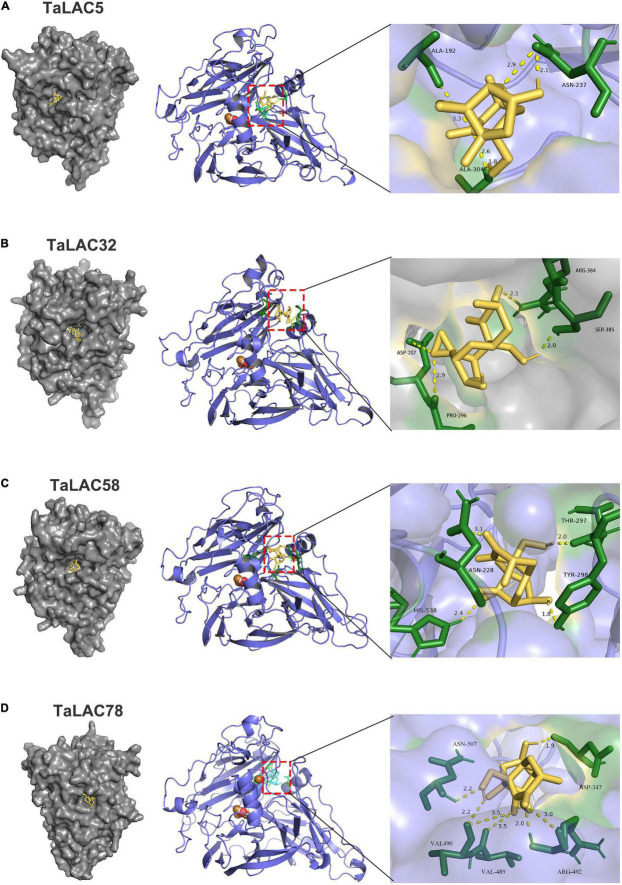
Molecular docking of TaLAC with DON. DON binding sites on the macromolecules of TaLAC5 **(A)**, TaLAC 32 **(B)**, TaLAC58 **(C)**, TaLAC78 **(D)**.

The range of substrates of LACs is broad due to their low substrate specificity. According to our previous analysis, most of the FHB-responsive TaLACs have lost T1Cu, whereas their basic 3D structures are complete and conserved. Molecular docking of TaLACs with lignin monomer indicated that TaLAC5, TaLAC32, and TaLAC58 can still coordinate sinapyl alcohol despite the loss of T1Cu (Supplementary Figures 5A–C). As a secondary metabolite with a small molecule, DON has the potential characteristics of being a ligand. Therefore, to understand whether DON can be bound by laccase and oxidized to reduce the toxicity of DON, we performed molecular docking to predict the binding mode of DON to TaLACs. The results indicated that DON can bind the protein of TaLAC5, TaLAC32, TaLAC58, and TaLAC78 (Figure 8). Moreover, the docking site of DON on both TaLAC5 and TaLAC58 are in the substrate pocket binding lignin monomer (Figures 8A,C). These results suggest that TaLAC might be able to trap DON and oxidize it.

## Discussion

As an important kind of multicopper oxidase, the LAC family has many homologs in plants and plays a pivotal role in various physiological processes, especially in biotic and abiotic stresses. Polyploidization, tandem duplication, and segmental duplication of wheat are the main causes for the expansion of *TaLAC* gene family members. Although 95 *TaLAC* genes were identified in wheat, only 14 specifically responded to FHB according to our data (Figures 1, 3 and Supplementary Figure 1). Unexpectedly, only TaLAC78 contains the complete activity center for oxidation, and the others lose the T1 copper ion ligand (Figure 5).

The absence of T1Cu may lead to the loss of oxidative activity for TaLACs. Considering the conserved function of LAC in lignin synthesis during the cell-wall formation process (Bao et al., 1993; Wang et al., 2015), the LAC without T1Cu may not have the ability to biosynthesize lignin, and thus may hinder the formation of secondary cell walls. During the process of *F. graminearum* invasion, the cell wall provides an important physical barrier (Paccanaro et al., 2017). Previous studies in cotton suggested that *GhLAC* gene enhanced resistance to *Verticillium dahliae*, mainly by increasing the lignification and lignin components of the cell wall (Hu et al., 2018; Zhang et al., 2019).

The prediction of the three-dimensional structure of the protein mainly depends on the reference template. When the aligned sequence is not completely consistent with the template sequence, it may also cause changes in the key domains. Although the key amino acids that bind the copper ion ligand in TaLAC1, TaLAC5, TaLAC11, TaLAC51, TaLAC58, and TaLAC74 are the same as the reference protein ZmLAC3 (Supplementary Figure 3), and the variation of amino acids flanking the key amino acids that bind T1Cu may also lead to the loss of T1Cu ions in the automated homology model (Figure 6 and Supplementary Figures 3, 4). However, this situation may not happen *in vivo* since the active centers of these LACs are still complete, which requires in-depth experimental verification.

As a hemibiotrophic pathogen, *F. graminearum* is in the initial biotrophic growth phase within 12 h post-inoculation (hpi) and then switches to necrotrophic growth phase to break the cell wall and invades into the plant cell to absorb nutrition from 36 hpi (Brown et al., 2010), when pathogen hyphae secretes a diverse portfolio of cell wall degrading enzymes to break through the plant cell wall (Wanyoike et al., 2002). TaLAC78 is the only LAC protein that has a complete oxidative activity center, which might play a pivotal role in cell wall protection. The expression patterns of *TaLAC78* in FHB-susceptible variety CS clearly show that *TaLAC78* starts accumulation from 1 DPI (Figure 7D), and reaches the peak transcript abundance at 3 DPI, when encounters the key time point of hyphae spread, predominantly inter- and intracellularly, in the pericarp parenchyma cells of the ovary and lemma tissues (Wanyoike et al., 2002). The hyphae reached the rachis at 5 DPI, which is the second peak of the *TaLACs* transcript levels (Figure 7D).

On the other hand, *TaLAC78* was slightly induced in SM, and the expression peak was delayed till 6 DPI (Figure 7D), and FHB symptom presents moderate in SM when compared with FHB-susceptible variety CS. The expression pattern of *TaLAC78* indicated that it may play a key role in the fungus-invading pathway by mediating the lignin biosynthesis to confer passive defense against *F. graminearum*. At the same time, it may act synergistically with TaLAC5 and TaLAC32 in toxin adsorption, since they showed similar expression patterns (Figures 7A,B,D).

*Triticum aestivum laccase* genes induced by *F. graminearum* suggested that these *TaLACs* might play positive roles in preventing hyphae expansion *via* protection of cell wall from being destroyed by fungus. However, we also found that *TaLAC51* and *TaLAC58* were inhibited by *F. graminearum* (Figures 1, 7C), suggesting that *TaLAC58* and its paralog might play opposite functions when comparing with the other *TaLACs*. Interestingly, *TaLAC58* was localized in a known QTL with a high confidence interval for Type II resistance to FHB, and SM carries a susceptible allele at the QTL according to our previous analysis (Li et al., 2016). In addition, it is possible that differences in expression patterns could be ascribed to the sequence variations in promoters of *TaLACs*, for instance, two important stress-related *cis-*elements in the promoter of *TaLAC58* are lost.

In the process of invading wheat spike, *F. graminearum* secretes DON, which penetrates the cell wall in preference to *F. graminearum* hyphae (Boenisch and Schäfer, 2011) and prevents the normal protein synthesis once entering the protoplast (Arunachalam and Doohan, 2013; Garreau de Loubresse et al., 2014; Chen et al., 2019). Existing studies showed that LACs can be localized in the cell wall (Hoffmann et al., 2020), and LACs may be also localized in the periplasmic space of cells because they are secreted proteins (Table 1). DON was localized in host cell walls, cytoplasm, chloroplasts, endoplasmic reticulum, ribosomes, as well as plasmalemma (Kang and Buchenauer, 2000). DON produced by fungus passes through the cell wall to enter the cell. In the periplasmic space, TaLACs may meet and trap DON, which would prevent DON from entering the cell to interfere with the normal metabolism of the cell. TaLACs may also further oxidize DON to less toxic forms, consequently alleviating the harm caused by DON to human and animal health; however, this attractive deduction needs further experimental validation.

## Conclusion

We identified 14 wheat *LAC* genes that respond to *F. graminearum* infection at the genome-wide level. The three-dimensional structure prediction showed that the oxidative active centers were incomplete for 13 of the 14 TaLAC proteins. Amino acid sequence alignment and three-dimensional reconstruction after amino acid *in silico* mutations indicated that their functions were postulated to be related to both the key amino acid variation that binds T1Cu and their flanking amino acids. Molecular docking prediction demonstrated that TaLACs that respond to FHB may work either by protecting the cell wall during *F. graminearum* infection or by trapping DON, or detoxifying DON to reduce the damage to cells. These *TaLACs* may be candidates for exploring the novel functions of TaLACs in wheat resistance to FHB.

## Data Availability Statement

The datasets presented in this study can be found in online repositories. The names of the repository/repositories and accession number(s) can be found below: https://www.ncbi.nlm.nih.gov/, PRJNA683746.

## Author Contributions

ZS conceived the idea and designed the experiments. YH and YZ performed the sample preparation and collection for RNA-sequencing. YH and NJ analyzed the data and performed the qRT-PCR validation. YZ performed the prediction of protein 3D structure model and molecular docking. SH performed the analysis of promoter and figure preparing. LL supported guidance for data analysis. ZS and TL wrote the manuscript. All authors read and approved the final manuscript.

## Conflict of Interest

The authors declare that the research was conducted in the absence of any commercial or financial relationships that could be construed as a potential conflict of interest.

## Publisher’s Note

All claims expressed in this article are solely those of the authors and do not necessarily represent those of their affiliated organizations, or those of the publisher, the editors and the reviewers. Any product that may be evaluated in this article, or claim that may be made by its manufacturer, is not guaranteed or endorsed by the publisher.

## Footnotes

1
https://www.arabidopsis.org/index.jsp

2
https://www.ricedata.cn/gene/

3
https://plants.ensembl.org//Triticum_aestivum/

4
https://www.uniprot.org/

5
http://pfam.janelia.org

6
http://bioinformatics.psb.ugent.be/webtools/plantcare/html/

7
http://202.194.139.32/expression/index.html

8
http://web.expasy.org/compute_pi/

9
https://services.healthtech.dtu.dk/

10
http://cello.life.nctu.edu.tw/

11
http://meme-suite.org/tools/meme

12
https://swissmodel.expasy.org/

13
http://zinc.docking.org/

## Supplementary Material

Supplementary Figure 1 Chromosomal distribution and tandem duplication of TaLACs across wheat genome. The tandem duplicated genes are marked by small boxes. The identified positions were then marked on the chromosomes using the MapChart tool, and the unit was Mb. Bold gene names present FHB responsive LACs.

Supplementary Figure 2 The expression patterns of 17 FHB responsive TaLACs under different stresses from fungus or PAMPs treatment. The Column charts were generated using public transcriptome data (http://202.194.139.32/expression/wheat.html). The original data come from the published papers of Zhang et al. (2014), Biselli et al. (2018), and Steuernagel et al. (2018).

Supplementary Figure 3 Amino acid sequence alignment of 14 TaLACs specifically responsive to FHB. The amino acids covered by red lines were involved in copper ions coordinating at T1, T2, or T3. The amino acids pointed by the triangle symbol is the key variant for coordinating T1Cu. Protein of *Zea mays* LAC3 (ZmLAC3) used as the reference. “*” indicates that the amino acid residues at the position are highly conserved across the 15 proteins. “:” indicates that the amino acid residues at the position are toward conservative across the 15 proteins and they have the same physicochemical properties. “.” indicates the amino acid residues at the position are semi-conservative.

Supplementary Figure 4 ZmLAC3 T1 copper ion was coordinated by 5 residues within 4Å. The amino acid residues coordinating with copper ion are shown in dashed lines representation.

Supplementary Figure 5 Molecular docking of TaLAC with sinapyl alcohol. The monolignol sinapyl alcohol binding sites on the macromolecules of TaLAC5 **(A)**, TaLAC32 **(B)**, TaLAC58 **(C)**, TaLAC78 **(D)**.
