# Supplementary figures and images for: Identification of Wheat LACCASEs in Response to Fusarium graminearum as Potential Deoxynivalenol Trappers
Source: Front Plant Sci. 2022 Mar 14;13:832800. doi: 10.3389/fpls.2022.832800 (PMC8964265; doi:10.3389/fpls.2022.832800)

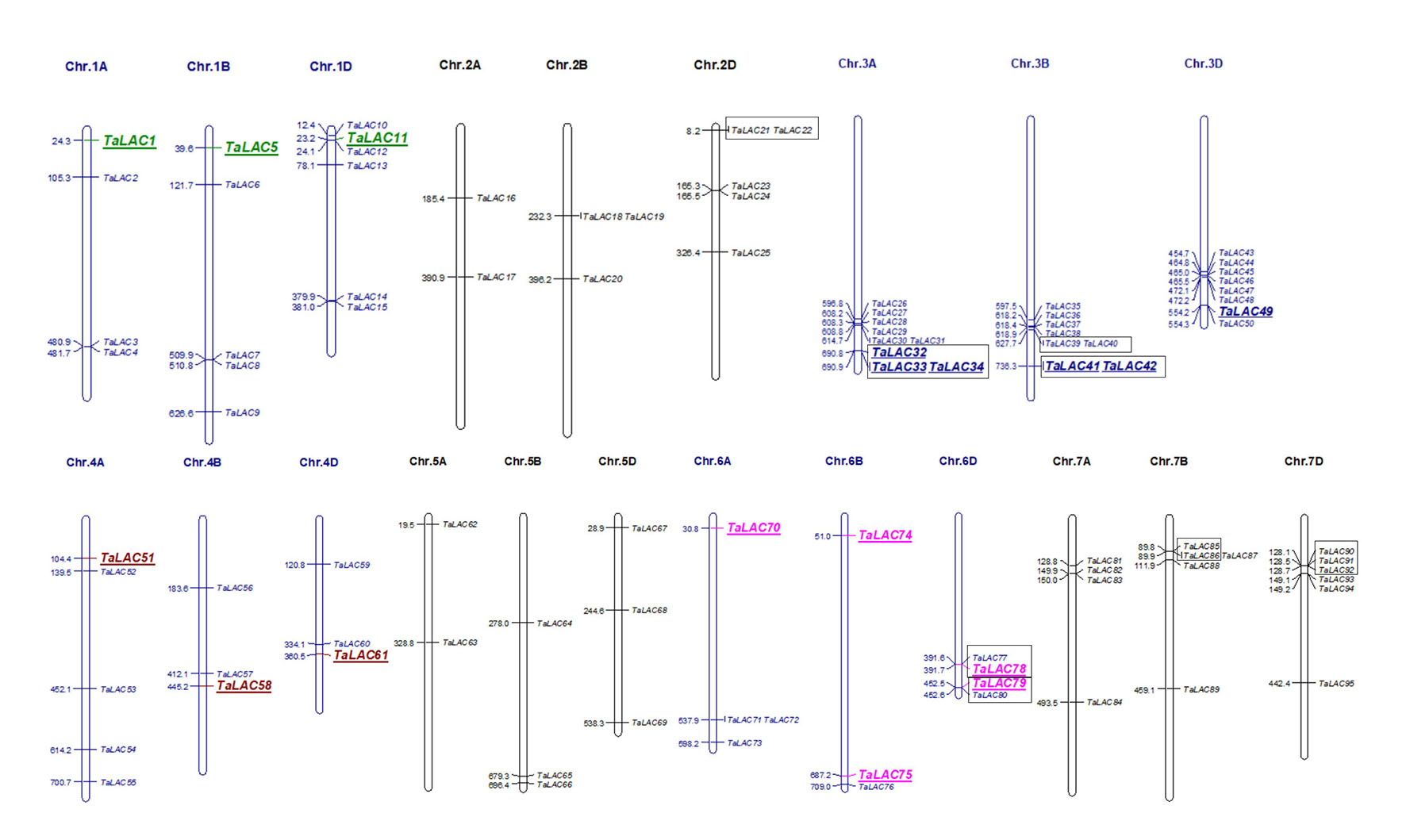

Supplement: Supplementary Figure 1 — Chromosomal distribution and tandem duplication of TaLACs across wheat genome. The tandem duplicated genes are marked by small boxes. The identified positions were then marked on the chromosomes using the MapChart tool, and the unit was Mb. Bold gene names present FHB responsive LACs. [file Image_1.JPEG]

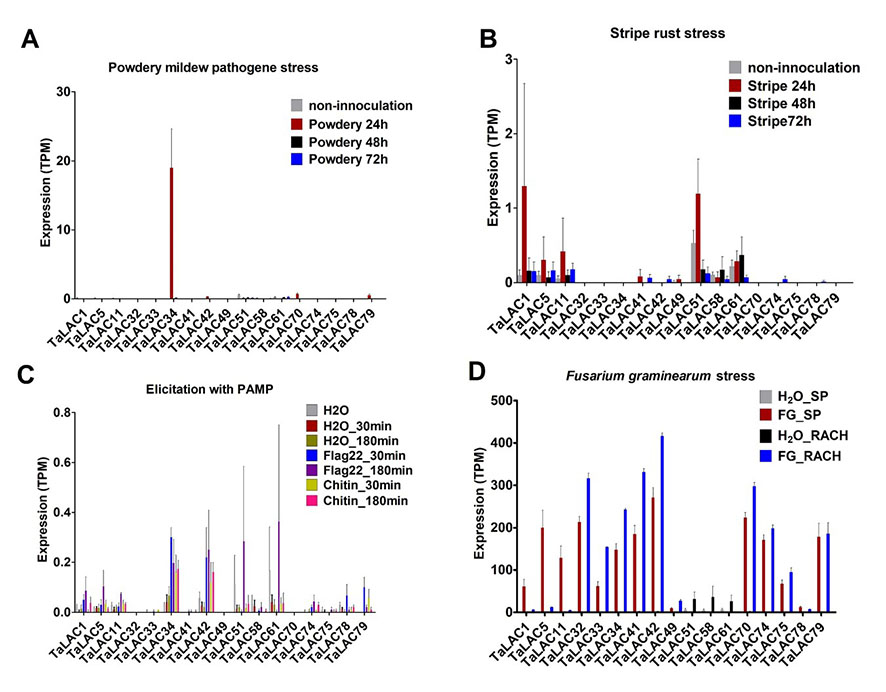

Supplement: Supplementary Figure 2 — The expression patterns of 17 FHB responsive TaLACs under different stresses from fungus or PAMPs treatment. The Column charts were generated using public transcriptome data (http://202.194.139.32/expression/wheat.html). The original data come from the published papers of Zhang et al. (2014), Biselli et al. (2018), and Steuernagel et al. (2018). [file Image_2.JPEG]

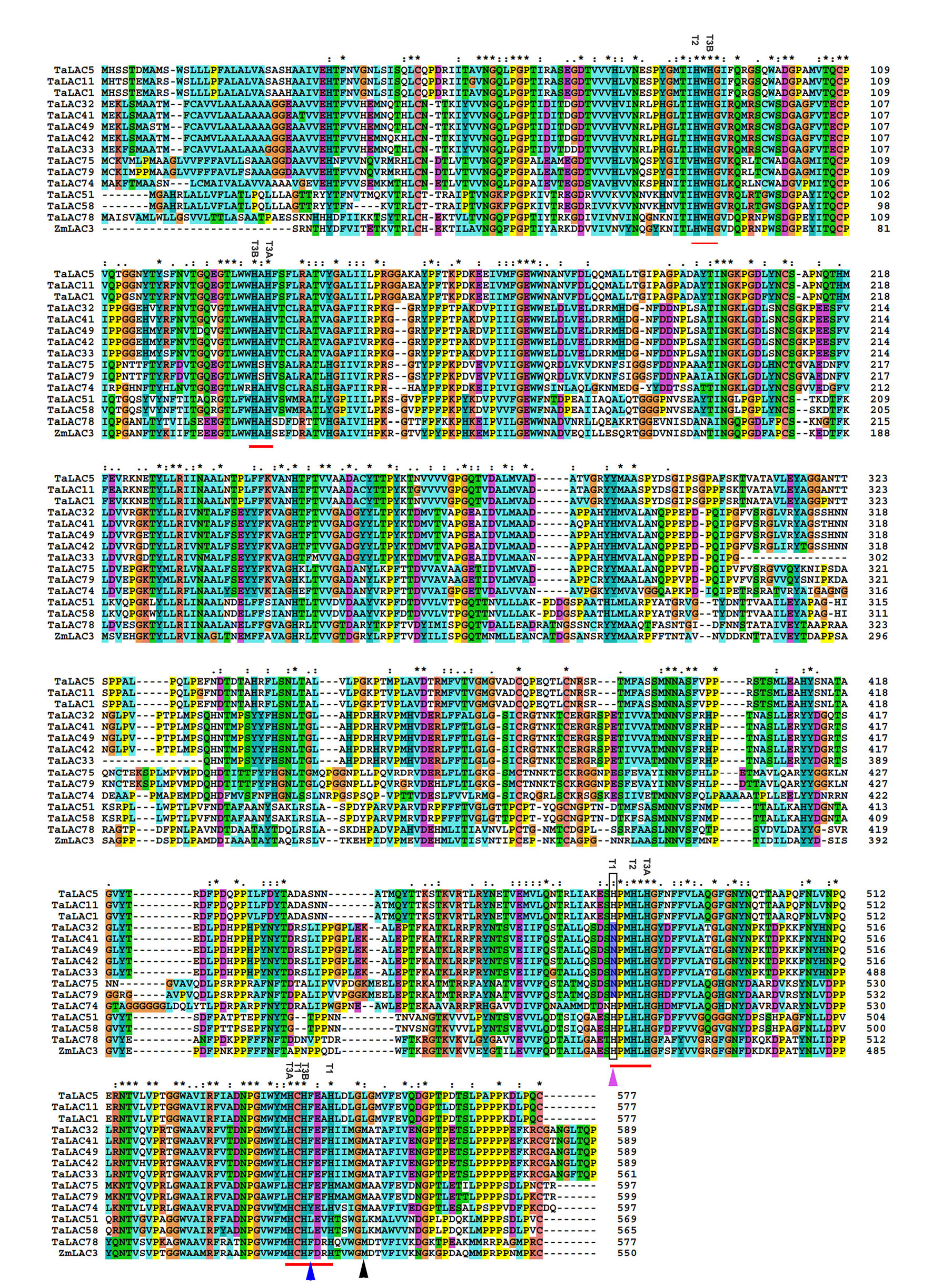

Supplement: Supplementary Figure 3 — Amino acid sequence alignment of 14 TaLACs specifically responsive to FHB. The amino acids covered by red lines were involved in copper ions coordinating at T1, T2, or T3. The amino acids pointed by the triangle symbol is the key variant for coordinating T1Cu. Protein of Zea mays LAC3 (ZmLAC3) used as the reference. “*” indicates that the amino acid residues at the position are highly conserved across the 15 proteins. “:” indicates that the amino acid residues at the position are toward conservative across the 15 proteins and they have the same physicochemical properties. “.” indicates the amino acid residues at the position are semi-conservative. [file Image_3.JPEG]

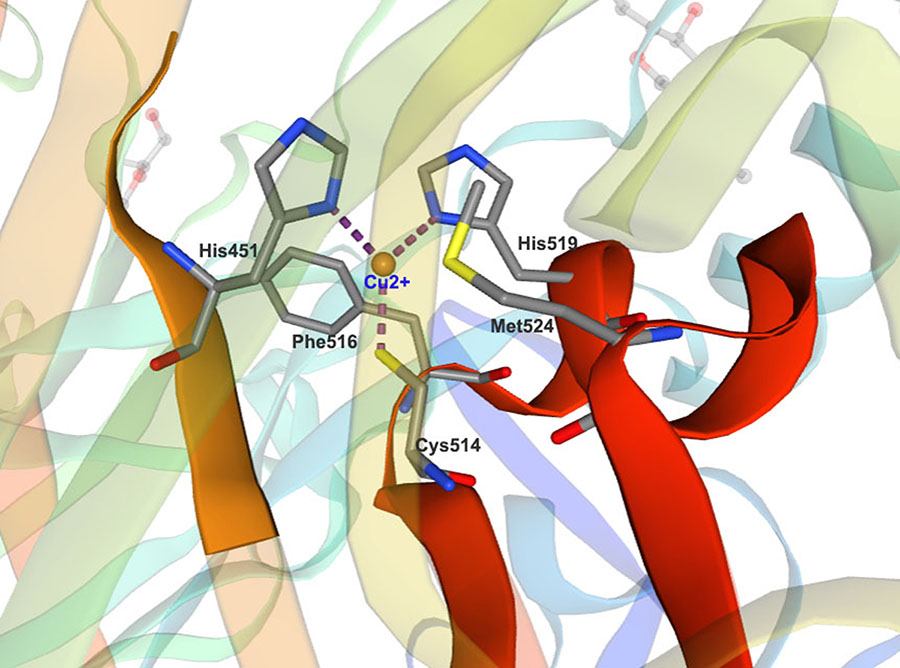

Supplement: Supplementary Figure 4 — ZmLAC3 T1 copper ion was coordinated by 5 residues within 4Å. The amino acid residues coordinating with copper ion are shown in dashed lines representation. [file Image_4.JPEG]

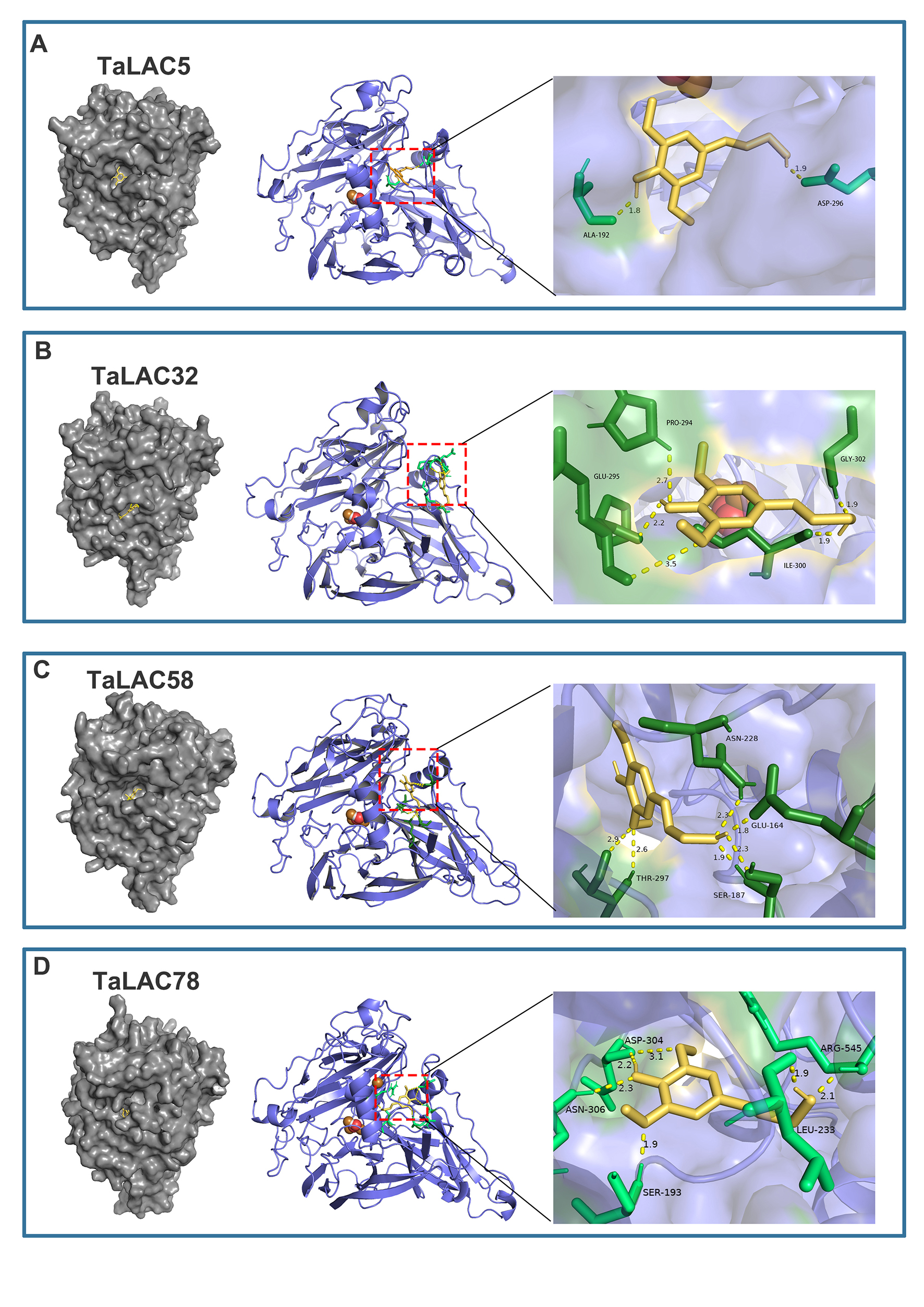

Supplement: Supplementary Figure 5 — Molecular docking of TaLAC with sinapyl alcohol. The monolignol sinapyl alcohol binding sites on the macromolecules of TaLAC5 (A), TaLAC32 (B), TaLAC58 (C), TaLAC78 (D). [file Image_5.JPEG]
